# High prevalence of potential drug interactions affecting mycophenolic acid pharmacokinetics in nonmyeloablative hematopoietic stem cell transplant recipients 

**DOI:** 10.5414/CP201884

**Published:** 2013-06-19

**Authors:** Alenka Jaklič, Carol J. Collins, Aleš Mrhar, Mohamed L. Sorror, Brenda M. Sandmaier, Meagan J. Bemer, Igor Locatelli, Jeannine S. McCune

**Affiliations:** 1 Department of Biopharmaceutics and Pharmacokinetics,; 2 Department of Social Pharmacy, University of Ljubljana, Ljubljana, Slovenia,; 3 Department of Pharmaceutics,; 4 Department of Medicine,; 5 Department of Pharmacy, University of Washington, and; 6 Clinical Research Division, Fred Hutchinson Cancer Research Center, Seattle, WA, USA

**Keywords:** mycophenolic acid, drug interactions, hematopoietic cell transplantation

## Abstract

Objective: Mycophenolic acid (MPA) exposure is associated with clinical outcomes in hematopoietic cell transplant (HCT) recipients. Various drug interaction studies, predominantly in healthy volunteers or solid organ transplant recipients, have identified medications which impact MPA pharmacokinetics. Recipients of nonmyeloablative HCT, however, have an increased burden of comorbidities, potentially increasing the number of concomitant medications and potential drug interactions (PDI) affecting MPA exposure. Thus, we sought to be the first to characterize these PDI in nonmyeloablative HCT recipients. Materials and methods: We compiled PDI affecting MPA pharmacokinetics and characterized the prevalence of PDI in nonmyeloablative HCT recipients. A comprehensive literature evaluation of four databases and PubMed was conducted to identify medications with PDI affecting MPA pharmacokinetics. Subsequently, a retrospective medication review was conducted to characterize the cumulative PDI burden, defined as the number of PDI for an individual patient over the first 21 days after allogeneic graft infusion, in 84 nonmyeloablative HCT recipients. Results: Of the 187 concomitant medications, 11 (5.9%) had a PDI affecting MPA pharmacokinetics. 87% of 84 patients had one PDI, with a median cumulative PDI burden of 2 (range 0 – 4). The most common PDI, in descending order, were cyclosporine, omeprazole and pantoprazole. Conclusion: Only a minority of medications (5.9%) have a PDI affecting MPA pharmacokinetics. However, the majority of nonmyeloablative HCT recipients had a PDI, with cyclosporine and the proton pump inhibitors being the most common. A better understanding of PDI and their management should lead to safer medication regimens for nonmyeloablative HCT recipients.

## Introduction 

Nonmyeloablative conditioning regimens for allogeneic hematopoietic stem cell transplantation (HCT) have expanded the availability of this procedure to patients who cannot tolerate the toxicity of high-dose conditioning due to age or comorbidity [1]. Approximately 75% of nonmyeloablative HCT recipients have pre-transplant comorbidities, as defined by the HCT-comorbidity index (HCT-CI), possibly increasing the number of potential drug interactions (PDI) [1]. A comprehensive review of PDI from postgrafting immunosuppression, which is administered for several months after allogeneic graft infusion, has yet to be conducted. An evaluation of PDI in nonmyeloablative HCT patients is imperative, especially considering the increased attention given to drug interactions in cancer patients receiving standard dose chemotherapy [2] and in solid organ transplant recipients [3]. 

Mycophenolate mofetil (MMF), an ester prodrug, is a key component of postgrafting immunosuppression after nonmyeloablative HCT. MMF is rapidly hydrolyzed to mycophenolic acid (MPA), its therapeutically active metabolite, by esterases in the gastrointestinal (GI) tract. MPA is a potent, reversible and non-competitive inhibitor of inosine monophosphate dehydrogenase (IMPDH) Types I and II, the inhibition of which blocks de novo purine synthesis in B and T lymphocytes [4]. After rapid absorption in the small intestine, MPA undergoes hepatic metabolism by various UDP-glucuronosyltransferase (UGT) isoenzymes to form MPA glucuronide (MPAG) [4]. Metabolites, of which MPA-7-O-glucuronide predominates, are excreted renally or into the bile via the ATP binding cassette transporter 2 (ABCC2, also multidrug resistance-associated protein 2 or MRP2) [4]. Metabolites can be converted back to MPA by the bacterial β-glucuronidase enzymes of the GI flora. The subsequent reabsorption of MPA as part of enterohepatic recycling (EHC) leads to a secondary peak in the MPA plasma concentration-time profile. HCT recipients infrequently exhibit a secondary MPA peak [5] and have reduced MPA plasma area under the concentration-time curves (AUCs) compared to solid organ transplant recipients [6]. 

Our group observed that low total MPA AUC was associated with a higher likelihood of graft rejection and low donor T-cell chimerism in nonmyeloablative HCT recipients [7]. High unbound MPA AUC was associated with a higher likelihood of cytomegalovirus (CMV) reactivation. PDI that decrease MPA AUC could increase the risk of graft rejection and low donor T-cell chimerism, while PDI that increase MPA AUC may increase toxicity. This led to our hypothesis that HCT patients are susceptible to drug interactions that affect MPA AUC, and that these PDI may be caused, in part, by concomitant medications administered for comorbidities unrelated to HCT. To evaluate this hypothesis, we compiled a comprehensive list of previously documented PDI affecting MPA AUC. We then used this list to conduct a study characterizing the cumulative PDI burden, defined as the sum of PDI for an individual patient over the first 21 days after allogeneic graft infusion. Because of the key role that MPA has as postgrafting immunosuppression for HCT recipients, we focused solely on PDI affecting MPA AUC and did not include drugs (e.g., acyclovir [8]) affected by MPA. 

## Methods 

### Literature review 

We sought to compile a comprehensive list of PDI and therefore evaluated several frequently used drug interaction databases and conducted literature searches in PubMed. The drug interaction databases evaluated were the University of Washington Drug Interaction Database [9], Stockley’s Drug Interactions [10], Lexicomp™ [11], Micromedex^®^ [12] and Drugs.com [13]. In addition, a PubMed search was conducted with the following terms: (i) mycophenolate OR (mycophenolic acid) AND interactions, (ii) mycophenolate OR (mycophenolic acid) AND (drug name), (iii) mycophenolate OR (mycophenolic acid) AND (drug name) AND interactions. 

### PDI affecting MPA pharmacokinetics 

PDI were categorized by the level of evidence and recommended management. The level of evidence was classified using the scale from Facts and Comparisons: Drug Interaction Facts™ [14]. Medications with level 5 scientific evidence (i.e., in vitro data only) were not further considered relevant due to the lack of pharmacokinetic data. The recommended action to manage a PDI was classified using Hansten and Horn’s operational classification of drug interactions (ORCA) [15]. ORCA classifies drug interactions on a 5-class scale: Class 1 is assigned to drug interactions that must be avoided at all times and Class 5 is assigned to those that can be ignored. The recommended action to manage a PDI was chosen by the first (AJ), second (CJC) and senior (JSM) authors, with a group discussion to handle any disagreements. Case reports were evaluated with the Drug Interaction Probability Scale (DIPS) [16]. The recommended action depended on whether the medication was related to the HCT procedure (i.e., an essential part of the postgrafting immunosuppression) or unrelated (i.e., medications to treat comorbidities or cancer-related syndromes or to alleviate toxicities). Calcineurin inhibitors, corticosteroids and antimicrobials were categorized as HCT-related. Proton pump inhibitors (PPIs) and valproate were classified as non-HCT related. 

### Patient population 

We evaluated concomitant medications in a cohort of nonmyeloablative HCT recipients who participated in a prospective biomarker study between November 2008 and February 2012. Written informed consent was obtained from all patients, and the study protocol was approved by the Institutional Review Board at the Fred Hutchinson Cancer Research Center (clinicaltrials.gov #NCT00764829). Oral MMF administration frequency and dose were specified by HCT clinical protocols. Patients received supportive care per institutional Standard Practice Guidelines as previously described [17]. 

### Study evaluating PDI 

All concomitant medications (both around-the-clock and “pro re nata”) were recorded weekly on standardized medication history worksheets. Medication doses were not collected. Concomitant medications were culled from the medication history worksheets by two independent raters, with discrepancies resolved by discussion and a third review. The number of concomitant medications and PDI were evaluated on Days 2, 7 and 21 after allogeneic graft infusion. These days were chosen because Days 7 and 21 were used in our prior pharmacodynamics analysis in nonmyeloablative HCT recipients [7]. A PDI was defined as the administration of a potentially interacting medication within 3 days before or on Days 2, 7 or 21. For each patient, the cumulative PDI burden over the first 21 days post HCT was calculated by adding the number of PDI on each day; each drug was counted only once. 

## Results 

84 patients were included in this retrospective analysis; characteristics are described in Table 1. 51 participants had concomitant medications recorded on all 3 days (i.e., Days 2, 7 and 21), 24 participants on 2 of the 3 days, and 9 participants on only 1 day. Patients took a median of 13 (range 7 – 24) medications including MMF and 87% patients had a PDI. The majority of PDI arose from HCT-related medications; these should be managed by monitoring (ORCA, Class 3) since there is no suitable alternative available. 

**Figure 1:**
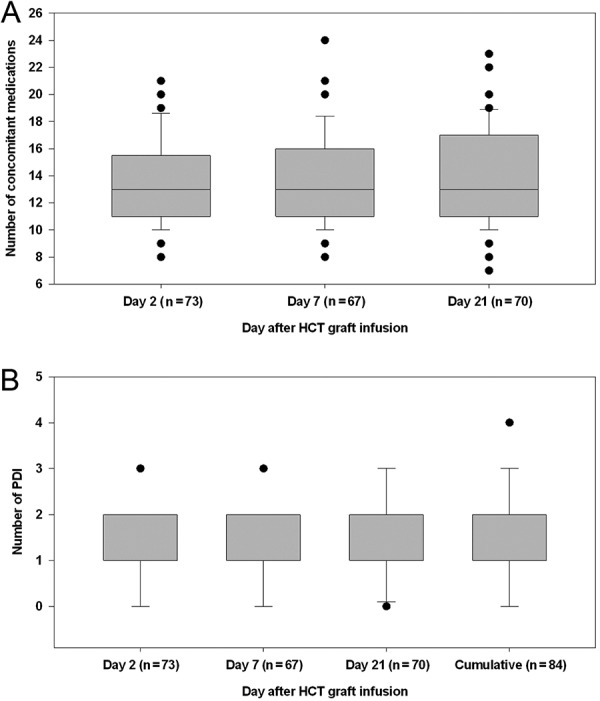
A: Concomitant medications per nonmyeloablative HCT recipients. B: Number of PDI per nonmyeloablative HCT recipients.

**Figure 2:**
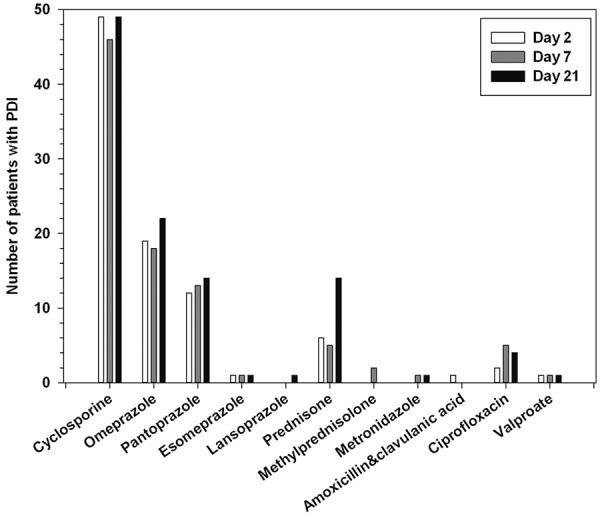
Time course of PDI.

Of 187 concomitant medications, 11 (5.9%) had a PDI. Figure 1 describes the number of concomitant medications and PDI, respectively. The median number of PDI per patient was 1 (Days 2 or 7) or 2 (Day 21), with a consistent range of 0 – 3 over all three occasions. The increased number of PDI on Day 21 was mostly due to increased corticosteroid administration. The median cumulative PDI burden was 2 (range: 0 – 4). Ten PDI were expected to decrease MPA AUC and one to increase MPA AUC (Table 2). The most common PDI were cyclosporine, omeprazole, and pantoprazole (Figure 2). 

### PDI decreasing MPA AUC 

Several PDI had the potential to decrease MPA AUC, including cyclosporine, corticosteroids, and PPIs. Decreasing MPA absorption or increasing MPA clearance would decrease MPA AUC, resulting in increased risk of graft rejection [7] or acute graft-versus-host disease (GVHD) [18]. Cyclosporine is often used as postgrafting immunosuppression with MPA. It inhibits ABCC2, thereby impairing EHC by inhibiting MPA reabsorption [18]. Cyclosporine is the only PDI previously reported in HCT recipients: the median MPA clearance was 33% higher in patients receiving concomitant cyclosporine compared to patients receiving tacrolimus [19]. The majority (n = 58) of our cohort received cyclosporine. Corticosteroids were predominantly used in this population to treat GVHD; their use increased from Day 2 to Day 21 (Figure 2). The potential effect of corticosteroids on MPA pharmacokinetics has been controversial; two studies reported no effect [32, 37], while another reported lower MPA exposure [20]. The majority of patients received prednisone, but 2 patients received methylprednisolone. PPIs potently inhibit gastric acid secretion, subsequently increasing the gastric pH. Higher gastric pH is expected to decrease MPA absorption by decreasing the release and hydrolysis of MMF [35]. Varying PPIs were used; omeprazole and pantoprazole predominated. Antibiotics may affect the EHC of MPA by impairing conversion of MPAG to MPA by GI bacterial β-glucuronidase. Patients took ciprofloxacin, metronidazole, and amoxicillin/clavulanic acid (Table 2). Notably, no patients took a fluoroquinolone and metronidazole at the same time. This combination has been shown to reduce MPA AUC by 33%, the most substantial effect seen in all antibiotic – MPA interaction studies [21]. 

### PDI increasing MPA AUC 

Valproic acid (level 3 evidence) was the only PDI to increase MPA AUC. Elevated unbound MPA AUC has been associated with more frequent CMV reactivation, therefore increasing toxicity [7]. The proposed mechanism for this interaction is inhibition of UGT2B7 enzymes, which would decrease metabolism of MPA to MPAG and increase MPA AUC. This is supported by case reports from 3 patients [22], leading to a DIPS score of 3. 

## Discussion 

To our knowledge, this is the first analysis of PDI in the setting of postgrafting immunosuppression in nonmyeloablative HCT recipients. Our key findings are that: 1) few (5.9%, 11 of 187) known concomitant medications have the potential to affect MPA pharmacokinetics based on current literature; 2) most patients (87%) had a PDI affecting MPA pharmacokinetics; and 3) cyclosporine, omeprazole and pantoprazole were the most common PDI. Only one other group has characterized PDI within HCT recipients [23]: 60% of 70 myeloablative HCT recipients had a PDI with an antibiotic during administration of the conditioning regimen [23]. We focused on MMF because it is administered daily for several months to nonmyeloablative HCT recipients, many of whom are either elderly or have comorbidities [1]. We targeted PDI affecting MPA pharmacokinetics because various pharmacodynamic studies suggest MPA AUCs or trough concentrations are associated with clinical outcomes in HCT populations [7, 24]. Furthermore, adverse outcomes are associated with drug interactions in solid organ and general medicine patients [25, 26], but no similar studies have been conducted in nonmyeloablative HCT recipients. Therapeutic drug monitoring of MPA is not standard of care in nonmyeloablative HCT recipients. We propose a PDI could be clinically significant if it may cause a ≥ 20% change in the total MPA AUC. This threshold was established based on the recent American Society of Blood and Marrow Transplantation report that generic immunosuppressants are considered interchangeable if their AUCs are within 20% of one another [27]. Previous groups considered a 66% reduction in MPA bioavailability [28] or a 10 mg/l×h change in MPA AUC [29] as relevant. Not surprisingly, there were also a varying number of publications regarding PDI (Table 2) with supportive data for some medications (e.g., PPIs) or conflicting information for others (e.g., corticosteroids). 

The majority of HCT recipients had at least one PDI affecting MPA pharmacokinetics, which confirms our hypothesis that these patients are susceptible to drug interactions due, in part, to concomitant medications administered for comorbidities unrelated to HCT. The HCT-CI of this patient population are comparable to those reported by Sorror et al. [1], who observed that an HCT-CI of 1 or greater is associated with worse survival in nonmyeloablative HCT recipients. Of the 187 concomitant medications, only a minority had a PDI (Table 2). Notably, only the PDI of the concomitant medications were evaluated; thus, not all previously reported drug interactions with MPA (e.g., rifampin [30], nonsteroidal anti-inflammatory agents [31]) were included. 

## Conclusion 

We found only a few concomitant medications had PDI that could affect MPA AUC. These medications, however, are commonly used as postgrafting immunosuppression in the HCT setting and potentially affect that majority of HCT recipients. Given the paucity of literature and the potential negative effects of PDI, especially in a population with multiple comorbidities, cross-sectional studies within a larger HCT population are needed to better comprehend PDI in nonmyeloablative HCT. Until these potential PDI are better understood, diligent review of concomitant medications is necessary to identify PDI affecting MPA pharmacokinetics, which could subsequently affect the therapeutic index of MMF. 

## Acknowledgments 

We are grateful to the study participants, their caregivers, and the patient care staff for their support of this study. This study was funded by National Institutes of Health grants HL091744 (JSM), HL36444 (BMS), CA78902 (BMS), CA18029 (BMS), and HL088021 (MLS). 

## Conflict of interest 

The authors declare no conflict of interest. 

**Table 1:** Characteristics^a^ of nonmyeloablative hematopoietic cell transplant (HCT) recipients.

Characteristic	No. of patients
Total no. of patients	84
Age (years)	61.7 (20.0-73.1)
Male	52 (62%)
Cancer diagnosis	
Non-Hodgkin lymphoma	28 (33%)
Chronic lymphocytic leukemia	16 (19%)
Acute myeloid leukemia	12 (14%)
Multiple myeloma	8 (10%)
Myelodysplastic syndrome	7 (8%)
Myeloproliferative disorders	4 (5%)
Acute lymphocytic leukemia	3 (4%)
Other	6 (7%)
HCT comborbidity index^b^	
0	8 (10%)
1 – 2	13 (15%)
3 – 4	31 (37%)
≥ 5	30 (36%)
End organ dysfunction^c^	
Renal dysfunction ^d^	11 (13%)
Liver dysfunction^e^	12 (14%)
Postgrafting immunosuppression concomitant with MMF	
Cyclosporine	58 (69%)
Tacrolimus	26 (31%)
Sirolimus and calcineurin inhibitor	13 (15%)

^a^Categorical data presented as number of participants meeting stated criteria; continuous data presented as median (min-max); ^b^HCT-Comorbidity index was assigned to 82 patients; ^c^1 patient, included in both values below, had both renal and liver dysfunction; ^d^creatinine clearance < 60 ml/min, calculated with Cockroft Gault equation using actual body weight; ^e^total bilirubin > than 2 times laboratory upper normal limits, alanine aminotransferase or aspartate aminotransferase > than 3 times laboratory upper normal limits.

**Table 2:** Overview of all PDI affecting MPA pharmacokinetics.

Drug^a^	Evidence per literature review	n^b^	Management^c^
↓ MPA area under the curve (AUC)^d^, ↓ efficacy			
Cyclosporine*	HCT [19], MPA clearance ↑ 33%	58	2
Proton pump inhibitors	Solid organ transplant (SOT), population pharmacokinetic (popPK) analysis [32], no effect		
Omeprazole	Healthy volunteer (HV) [33], MPA AUC ↓ 23%	28	2
Pantoprazole	Autoimmune disorders (AID) [34], MPA AUC ↓ 37%; SOT [35], MPA AUC ↓ 27%	20	2
Esomeprazole	Assumed similar to omeprazole [33]	1	2
Lansoprazole	SOT [36], MPA AUC ↓ 25%	1	2
Corticosteroids*	SOT [20, 32, 37], conflicting data		
Prednisone	SOT, conflicting data with no effect [37, 38] or lower MPA exposure [20]	15	3
Methylprednisolone	SOT [20], MPA clearance ↓ 25%	2	3
Antibiotics	SOT, PopPK study [32], no effect		
Metronidazole	HV [21], MPA AUC ↓ 19%	1	3
Amoxicillin/clavulanic acid	SOT, MPA Ctrough ↓ 46% [39]; SOT, case report^e^ [40]	1	3
Ciprofloxacin	SOT, MPA Ctrough ↓ 46% [39]; HCT, case report^e ^[41]	7	3
↑ MPA AUC, ↑ risk for toxicities			
Valproate^e^	SOT, case report [22]	1	2

^a^HCT medications are astericked; ^b^each PDI was counted once per patient over the entire study period; ^c^ORCA [15] classification of drug interactions with 2 (usually avoid combination: use only under special circumstances) and 3 (minimize risk: assess risk and take recommended actions including considering alternatives, circumventing or monitoring); ^d^level 2 scientific evidence [14]; ^e^level 3 scientific evidence [14].
